# Supplementary material for: Repurposing Sulfasalazine as a Radiosensitizer in Hypoxic Human Colorectal Cancer
Source: Cancers (Basel). 2023 Apr 18;15(8):2363. doi: 10.3390/cancers15082363 (PMC10137052; doi:10.3390/cancers15082363)
Supplement: Supplementary file 1 [file cancers-15-02363-s001.zip › cancers-2331462-supplementary.pdf]

Supplementary files

Materials & Methods:

1. MTT assays

The cytotoxicity of SSZ was determined by MTT assay. Briefly, cells were grown to sub-confluency in 96-well plates and treated with different concentrations of SSZ overnight. Hereafter, medium was aspirated and 50µL of MTT reagent (0.5mg/mL) was added to the cells for 1.5hours. 200µL MTT solvent (19:1 DMSO/HCl) was added to dissolve the formazan crystals inside the cells. Absorbance was measured at a wavelength of 540nm by a spectrophotometer (Bio-Rad Laboratories, Temse, Belgium). Cell viability was determined by dividing the absorbance of the treated cells to that of the untreated cells.

2. Kinetic growth assay

The influence of SSZ on the cell growth was determined by following up the confluency of cells in real-time using the Incucyte live cell imager (Essen Biosciences, Royston, UK). Shortly, cells were grown in 96-well plates and treated with SSZ when they reached sub-confluency. The confluency was measured with the Incucyte software (Incucyte ZOOM 2018A, Essen Biosciences, Royston, UK) for at least 68h.

3. Seahorse Metabolic profiling

The Seahorse XF96 analyzer (Agilent Technologies, Santa Clara, CA, USA) was used for determining the oxygen consumption rate (OCR) and extracellular acidification rate (ECAR). In short, $2.5x10^4$ cells were seeded in 96-well plates. Treatment with SSZ happened overnight. Afterwards, cells got equilibrated with unbuffered Dulbecco's Modified Eagle Medium (DMEM) supplemented with 2mM glutamine and 10mM glucose at pH 7.4. Cells were put in a 37°C and CO_2-free incubator. After this incubation period, cells were put in the Seahorse analyzer, and specific inhibitors were sequentially added: Oligomycin (1µM), FCCP (1µM) and rotenone/Antimycin A (0.5µM). OCR levels were normalized to the protein content of each condition.

4. Western Blot

Cell lysates were made by adding a 1% Triton-X lysis buffer to the cells, complemented with a phosphatase inhibitor, protease inhibitor, and leupeptin. Protein concentrations were determined, and equal amounts of protein were loaded on a gel containing 12% polyacrylamide. Nitrocellulose membranes (ThermoFisher Scientific, Merelbeke, Belgium, 88018) were used for an overnight protein transfer at 4 °C. Afterward, blocking of the membranes was conducted with 5% BSA in TBS. Incubation of membranes with primary antibodies was achieved overnight at 4 °C. Near-infrared secondary antibodies (IRDyes 680 RD or 600 CW, Li-Cor Biosciences, Bad Homburg, Germany) were used for labeling primary antibodies. The Odyssey Fc Imaging System (LI-COR, Biosciences, Bad Homburg, Germany) was used for visualization of the signal. The following primary antibodies were used: anti-xCT (Cell Signaling Technology, Leiden, The Netherlands, 12691S) and anti-α-tubulin (Sigma Aldrich, Antwerp, Belgium, T9026).

Figures:

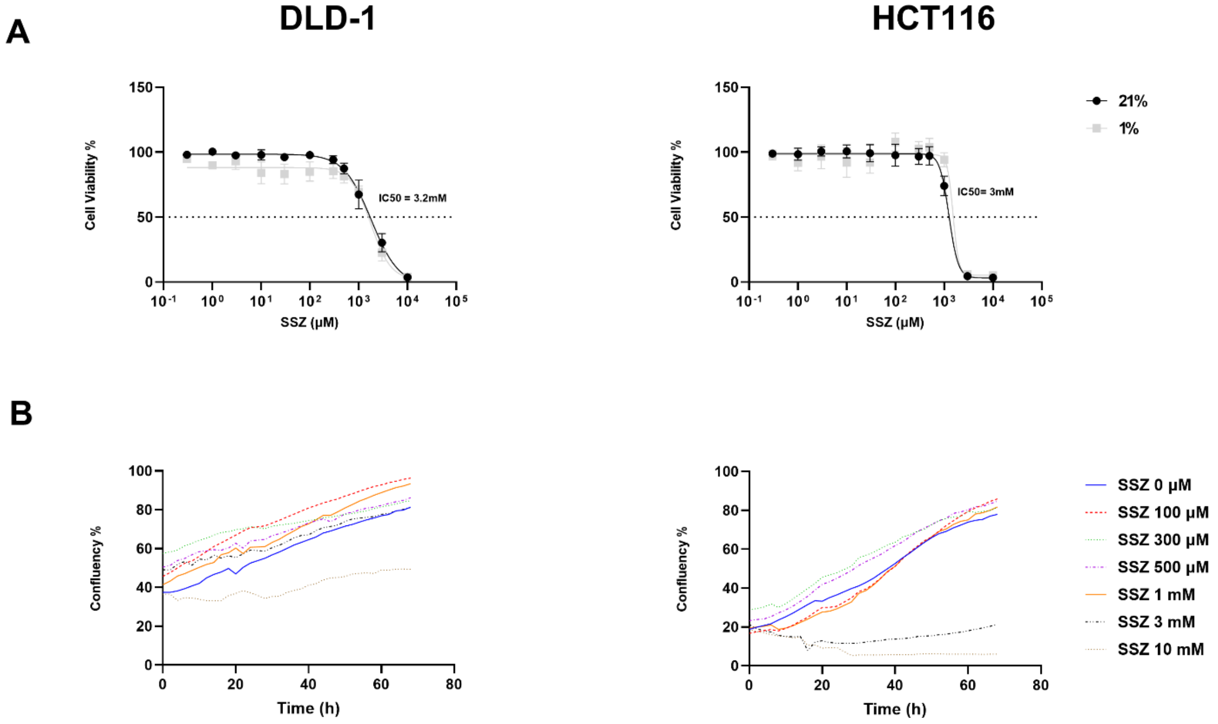

Figure S1. SSZ induces a dose-dependent toxicity in human colorectal cancer cells. DLD-1 and HCT116 were treated with SSZ (16h) at indicated concentrations. **(A)** Cell viability was determined under normoxic (21%) and hypoxic (1%) conditions by an MTT assay in DLD-1 (left) and HCT116 (right) cell lines. **(B)** Cell growth was also followed up in real-time after SSZ treatment (16h) with the Incucyte ZOOM for DLD-1 (left) and HCT116 (right) cell lines for 68h. Data are represented as mean ± SEM (for MTT) and as median (for Incucyte experiment), *n*>3.

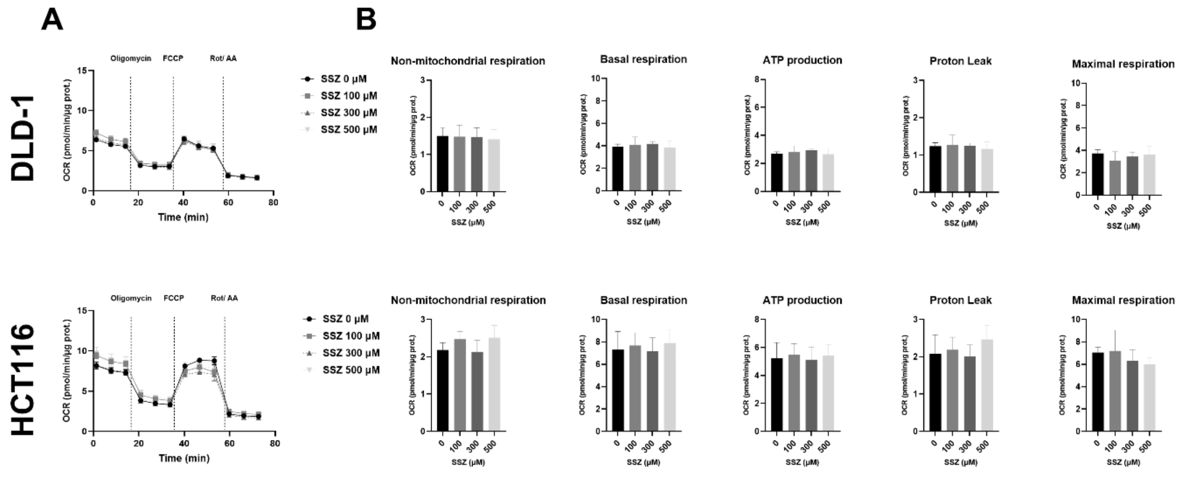

Figure S2. xCT inhibition does not influence the mitochondrial metabolism of human colorectal cancer cells. CRC cells were treated with SSZ (16h) and the influence on the mitochondrial metabolism was determined by a MitoStress Test, as measured with a Seahorse analyzer. **(A)** Influence of SSZ treatment on the mitochondrial metabolism of DLD-1 (top) and HCT116 (bottom) cell lines. **(B)** Summarizing graphs of the representative figure in A, showing all the calculated

parameters in DLD-1 (top) and HCT116 (bottom) cell lines. Data are represented as mean ± SEM or as a representative experiment, n= 2.

A

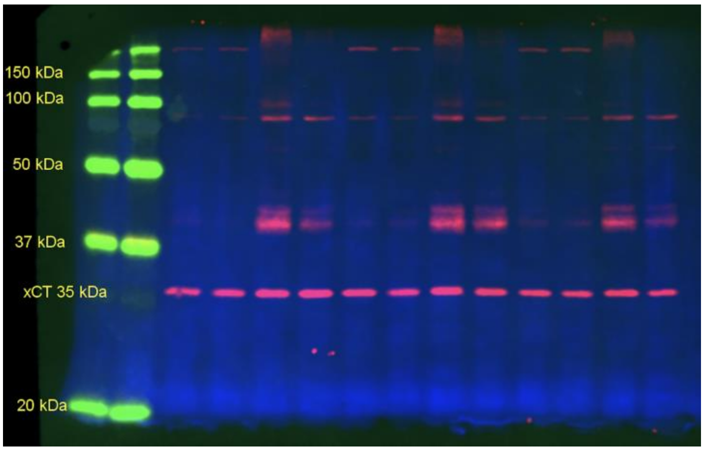

B

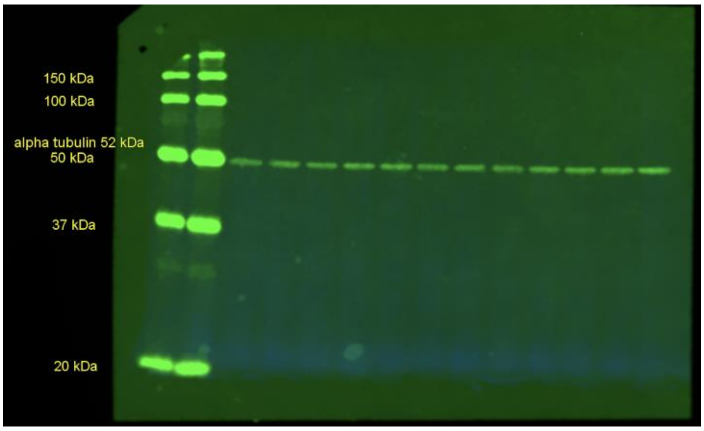

Figure S3. Uncropped Western Blot shown in Figure 1E. **(A)** Uncropped Western blot showing xCT expression in DLD-1 (band 1-2) and HCT116 (band 3-4) under normoxic (band 1-3) and hypoxic (band 2-4) conditions. **(B)** Uncropped Western blot showing alpha tubulin expression in DLD-1 (band 1-2) and HCT116 (band 3-4) under normoxic (band 1-3) and hypoxic (band 2-4) conditions.
